# Hints on the Subject of Artificial Teeth and Plates

**Published:** 1850-10

**Authors:** J. W. Crane

**Affiliations:** New York


### ARTICLE VIII.

### *Hints on the subject of Artificial Teeth and Plates.*

Mr. Editor:

*Dear Sir:*—Will you permit me to contribute a few items, for the benefit of students and young practitioners of dental surgery, on the subject of artificial teeth and plates. I have examined a number of works on this subject, and have come to the conclusion that authors have not written all they know on this subject, or they have not come up to the latest improvements in mechanical dentistry.

The first thing of importance I shall speak of, is the use of clasps.

My experience the last five or six years, has proved beyond a doubt, that they are used in many cases where there is not the least necessity for them, and to the destruction of one or more teeth.

I have seen three or four teeth confined by clasps in a mouth, where it was not necessary to use one. If our teeth are valuable in proportion to their number, then the information which I propose to give, will be acceptable to those who have not learned by experience, the true plan of preserving teeth from this unnecessary appendage.

There are certain circumstances where the clasp is indispensable, but experience has taught us that teeth can be inserted where there are many valuable teeth standing in the same jaw, without the use of clasps. They are also as useful in mastication, as those inserted on a clean gum, where every tooth has been extracted.

The following cases will illustrate the truth of this assertion, and can be inspected by any respectable member of the profession.

Miss P. has seven front inferior teeth of her own, the back teeth are inserted without clasps.

Mr. R. has three inferior incisors, two cuspidati, one bicuspid and one dens sapientiæ, the others in the lower jaw, are artificial and used without clasps, he has also fourteen superior artificial teeth and no springs. Mrs. M. has three upper molars, and ten artificial teeth in front of them without clasps.

Mrs. —— has one superior front incisor, one lateral, two canine, the others are artificial and used without clasps. Mr. A. has lost all of his upper teeth, except six in front, the molars and bicuspids are artificial and used in mastication without clasps.

Mrs. W. has ten gum teeth inserted in front of her upper molars, in the same way.

If the above should be acceptable, I shall next give my experience on the absurdity of using wide plates, for the purpose of adhesion, also the ridiculous large air chamber in the roof of the mouth, so much in vogue in this city. Another subject of great importance in dental surgery should be corrected, viz. the filling of the six years old molars after the decay is very deep and near the nerve.

J. W. CRANE.

New York, *Jan'y 10th,* 1850.

Note.—The deleterious effects produced upon natural teeth by the application of clasps, is a subject to which the attention of many of the members of the profession have been directed for several years, and their employment has been much less frequent during the last four or five years than formerly. In vol. ix, No. 4, page 357, there is a well written article upon this subject, by Dr. P. H. Austin, and another by Dr. W. H. Dwinelle, in vol. x, No. 2, page 108, of the same journal. The attention of the profession is also directed to the subject in the American edition of Fox on the Human Teeth, and in the editor's Principles and Practice of Dental Surgery. Indeed, to such perfection, has the suction or atmospheric principle of applying artificial teeth been brought, that many practitioners dispense with the use of clasps in almost every case.

The injury to the natural teeth, is not, as many suppose, produced by the mechanical action of the clasps; it is the result of the chemical action of the secretions of the mouth retained between them and the organs to which they are applied. When the employment of clasps, therefore, becomes indispensable, the patient should be apprised of the importance of removing the piece several times a day, and thoroughly cleaning the teeth to which the clasps have been applied; also, of leaving the piece out of the mouth during the hours of rest. The injury liable to be produced is always in proportion to the softness of the texture of the teeth, and the unhealthy condition of the secretions of the mouth.—*Editor.*

